# Anti-HEV seroprevalence and rate of viremia in a German cohort of dogs, cats, and horses

**DOI:** 10.1038/s41598-023-46009-y

**Published:** 2023-11-07

**Authors:** S. Pischke, E. V. Knoop, M. Mader, L. Kling, A. Wolski, A. Wagner, K. Mueller, T. Horvatits, J. Stiller, K. Wisnewski, B. Kohn, J. Schulze zur Wiesch, M. H. Groschup, M. Eiden

**Affiliations:** 1 Department of Medicine, University Medical Center Hamburg-Eppendorf, Hamburg, Germany; 2 German Center for Infection Research (DZIF), Hamburg-Lübeck-Borstel and Heidelberg Partner Sites, Hamburg, Germany; 3 SYNLAB.Vet GmbH, Berlin, Germany; 4 Vetambulanz Hamburg, Hamburg, Germany; 5 Small Animal Clinic, School of Veterinary Medicine, Freie Universität Berlin, Berlin, Germany; 6 Small Animal Clinic, University of Leipzig, Leipzig, Germany; 7 Institute of Novel and Emerging Infectious Diseases, Friedrich-Loeffler-Institut, 17493 Greifswald-Insel Riems, Germany

**Keywords:** Microbiology, Gastroenterology

## Abstract

Hepatitis E virus (HEV) genotype 3 infections in Germany are mainly transmitted zoonotically through the consumption of swine meat. Furthermore, there is evidence that pets might come into contact with HEV, but the relevance of companion animals as possible sources of HEV transmission in Germany still needs to be defined. A monitoring study was therefore carried out on dogs, cats, and horses from Germany. In total 365 serum samples from pets (124 dogs, 119 cats, and 122 horses) were tested for HEV by PCR and for anti-HEV antibodies by a commercial ELISA. The HEV seroprevalence determined by the sero-assay varied significantly between dogs (10%), cats (6%), and horses (2%). Liver injury-related enzymes, alanine transaminase (ALT), and aspartate transaminase (AST) showed no differences between HEV-positive or negative animals. None of the pet serum samples tested positive for PCR. This serological study suggests that dogs and cats are significantly exposed to HEV in Germany, while horses are of minor relevance.

## Introduction

Hepatitis E virus (HEV) infections occur worldwide^1^. In tropical developing countries human-associated HEV genotypes 1 and 2 (HEV-1/2) of the genus *Paslahepevirus* are mostly transmitted through contact with contaminated drinking water leading to epidemic outbreaks. In contrast, genotypes 3 and 4 (HEV-3/4) of the same genus are mostly transmitted zoonotically in industrialized countries mainly from wild boar and pig to humans through ingestion of undercooked meat^1^. Furthermore, rat-derived HEV strains (HEV-C1) from the genus *Rocahepevirus* are of particular importance^2^ as cases of human infections caused by this variant have been diagnosed in Hong Kong and Spain^3^. Although numerous studies highlighted the role of the natural reservoir hosts especially pig and wild boar but also deer and rabbits on zoonotic transmission of HEV, significantly fewer studies have investigated the relevance of pets as possible hosts and potential sources of infection (Table 1). In general, numerous viruses that can infect companion animals are also infectious to humans^4^. This raises the question of the significance of HEV infections in companion animals. An in silico analysis of host genetics and HEV genetics identified dogs, and rats as potentially susceptible to *Paslahepevirus* infections, while cats and dogs were described as susceptible to HEV-C1 infections^5^. To get more information on HEV infections in German cats, dogs, and horses, a molecular and serological analysis of serum samples provided by veterinary diagnostic laboratories was conducted.

**Table 1:** Previous studies concerning the relevance of HEV and anti-HEV positivity in dogs, cats, and horses (chronological order).

Author	Year	Sample type	Result: rate of positivity (absolute numbers)	Technique	Country of origin	References
Arankalle	2001	Dogs, blood	23% (10/44)	Anti-HEV, in-house assay	India	^17^
Okamoto	2004	Cats, blood	33% (44/135)	Anti-HEV, in-house assay	Japan	^13^
			0% (0/135)	PCR		
Vitral	2005	Dogs, blood	7% (3/43)	Anti-HEV, in-house assay	Brazil	^18^
Mochizuki	2006	Cats, blood	2% (4/202)	Anti-HEV, in-house assay	Japan	^11^
		Dogs, blood	0% (0/424)			
Saad	2006	Work horses, blood	13% (26/200)	Anti-HEV, in-house assay	Egypt	^19^
			4% (4/100)	PCR		
Christensen	2008	357 human blood donors	Contact with horses is a risk factor for anti-HEV IgG positivity	Serology in humans (NIH-assay)	Denmark	^20^
Peralta	2008	Cats, blood	11% (6/54)	Anti-HEV, in-house assay	Spain	^12^
Zhang	2008	Horses, blood	16% (8/49)	Anti-HEV, in-house assay	China	^21^
		Dogs blood	18% (21/101)	PCR		
		Horses, blood	2% (1/49)			
		Dogs, blood	0% (0/101)			
Liu	2009	Dogs, blood	12% (23/192)	Anti-HEV, Wantai, and Dot-blot assays	China	^22^
Shao	2009	Dogs, bile specimens	0% (0/178)	PCR	China	^23^
Song	2010	Cats, blood	8% (8/99)	Anti-HEV, in-house assay	Korea	^24^
		Dogs, blood	0% (0/213)			
Dong	2011	Dogs, blood	1% (2/212)	Anti-HEV, in-house assay	USA	^25^
Geng	2011	Horses, blood	14% (40/280)	Anti-HEV, Wantai assay	China	^26^
Mesquita	2014	373 veterinarians, blood	Pet veterinarians have no increased risk for HEV exposure	Anti-HEV, Wantai assay	Portugal	^27^
McElroy	2015	Dogs, blood	1% (2/247)	Anti-HEV, in-house assay	United Kingdom	^28^
Wang	2016	Dogs, blood	19% (84/442)	Wantai-assay	China	^29^
			0% (0/442)	PCR		
Yonemitsu	2016	Dogs, blood	< 0.6% (1/170)	Anti-HEV, in-house assay	Japan	^30^
		Cats, blood	12% (2/17)			
Dähnert	2017	Dogs, blood	57% (47/83)	Anti-HEV, Wantai assay	Germany	^8^
		Cats, blood	32% (21/65)			
Zeng	2017	Dogs, blood	37% (1641/4490)	Anti-HEV, Wantai assay	China	^31^
Mooij	2018	Humans, blood	34% of blood donors without dog contact and 30% with dog contact serologically positive	Wantai-assay	Netherlands	^32^
Garcia-Bocanegra	2019	Horses, blood	0.4% (3/692)	PCR	Spain	^33^
Lyoo	2019	Dogs, blood	28% (81/287)	Anti-HEV, Wantai assay	South Korea	^34^
		Veterinarians, blood	5% (2/40)			
Li	2020	Dogs, blood	19% (30/162)	Anti-HEV, Wantai assay	Netherlands	^10^
		Cats, blood	15% (7/47)			
		Horses, blood	18% (4/22)			
Veronesi	2020	Dogs, blood	38% (32/84)	Anti-HEV, Wantai assay	Switzerland	^35^
Capozza	2021	Dogs, blood	3% (10/324)	Anti-HEV, Wantai assay	Italy	^9^
Bernadini	2022	Dogs, serum	5% (4/80)	Anti-HEV, DIA.PRO assay	Italy	^36^
		Dogs, rectal swabs	0% (0/80)	PCR		
Caballero-Gomez	2022	Cats, blood	3% (4/144)	MP assay for veterinary use	Spain	^7^
		Dogs, blood	10% (15/152)			
Li	2022	Cattle, cats, dogs, blood	Cattle, cats, dogs, swine and rats are possible hosts	Codon analysis identified possible HEV hosts	China	^5^
Yoon	2022	Horses	12% (35/283)	Anti-HEV, Wantai assay	Korea	^37^
			0% (0/100)	PCR		

## Material and methods

### Sampling

All samples were obtained from a routine veterinary laboratory (Synlab, Berlin, Germany). All samples have been collected in April or May 2022. Only serum samples from which sufficient material for PCR and serology (approx. 1 ml) was available after routine diagnostic could be tested. Initially, the goal was to test 120 dogs, 120 cats and 120 horses, but due to availability of samples there were small variations (dogs n = 124, cats n = 119, horses n = 122). Initially, we had tried to achieve an even distribution between animals with elevated liver values and those without elevated liver values in the first 200 or so animals, but since this was not realistically achieved, we switched to unselected serum samples regardless of how high the liver values were.

Basic characteristics such as sex, age, breed, or serum concentrations of enzymes aspartate transaminase (AST) and alanine aminotransferase (ALT) were provided in the far majority of studied pets. Initially, it was tried to include equal numbers of samples with elevated and normal liver values, but this was not successful so we switched to "unselected" study subjects.

No experiments on vertebrates were performed as part of this study. Only retrospective serum samples obtained for diagnostic purposes were retrospectively analyzed.

Therefore, the ARRIVE guidelines do not apply to the study.

The datasets generated and/or analyzed during the current study are not publicly available, because the data were examined completely anonymously and public access would allow individual authors of this manuscript to identify the animals and their serological status. However, the data are available from the corresponding author on reasonable request.

Due to anonymized testing, none of the authors of this paper can currently assign the serological results to individual animals with names and owners, and this anonymity should be preserved as far as possible.

### Completeness of the dataset

There were 2555 data collected for the entire study cohort, of which 484 (19%) were missing. In detail, the following were present: anti-HEV IgG value at 100%, OD value at 100%, AST at 90%, ALT at 85%, sex 68%, age 63%, race 62%.

### Serology

Serological testing has been performed by the MP Diagnostics HEV ELISA 4.0 (MP Biomedicals Germany GmbH, Eschwege), a commercial test able to detect anti-HEV in mammals.

Since the serum samples were completely anonymized, the personal data of the animal owners were not known during the testing. Therefore, no written consent of the animal owners is available. However, this is also not necessary. According to German laws, there is an obligation to obtain consent and an ethical vote or animal ethical vote for a prospective study and retrospective analyses, but not for retrospective anonymized studies. After consultation with the Ethics Committee of the Hamburg Medical Association, a formal ethics vote is not required for retrospective, anonymized serum analyses.

### Molecular biology

All animals have been tested by the RealStar^®^ HEV RT-PCR Kit 2.0 (Altona Diagnostics, Hamburg Germany). Serological positive serum samples have been re-tested by a second independent SYBR Green-based nested in-house broad range reverse-transcriptase (RT-) PCR, which targets a highly conserved region of the RNA-dependent RNA Polymerase within the ORF1 covering all genera of the subfamily *Hepevirinae* including genus *Rocahepevirus*^6^.

### Statistical analysis

Continuous variables with a non-normal distribution were expressed as median and interquartile range (IQR). Groups were compared using the Mann–Whitney *U* test. Categorical variables were expressed as a number (%) and compared with Fisher’s exact test. p values less than 0.05 were considered statistically significant. Statistical analyses were performed using SPSS, version 21.0 (IBM Corp., Armonk, NY, USA).

## Results

In total 365 serum samples of companion animals have been studied. In 246/365 (67%) the sex was known and 55% of these were male (n = 135). In 230/365 the age was known (63%), in 328/365 the AST was known (90%) and in 309/365 the ALT was known (85%) Characteristics of anti-HEV positive and negative animals are depicted in Table 2.

**Table 2:** Comparison of anti-HEV positive and negative animals*.

		Anti-HEV positive	Anti-HEV negative
Dogs (n = 124)	Sex	7 male/3 female/2 unknown	44 male/31 female/37 unknown
	Age in years, mean (range, Std. dev.)	9.9 (7–14, 2.6)	10.2 (0–17, 3.6)
	AST (normal < 62 U/l) in U/l (range, Std. dev.)	89.8 (30–221, 76.1)	126.9 (11–1280, 197.9)
	ALT (normal < 118 U/ml)in U/l (range, Std. dev.)	89.8 (30–221, 76.1)	404.6 (100–1525, 479.3)
Cats (n = 119)	Sex	3 male/3 female/1 unknown	49 male/42 female/21 unknown
	Age in years, mean (range, Std. dev.)	15.0 (14–16, 1.4)	11.5/0–20, 4.9)
	AST (normal < 47 U/l) in U/l (range, Std. dev.)	133.6 (21–361, 152.5)	181.9 (8–6744, 660.6)
	ALT (normal < 102 U/l) in U/l (range, Std. dev.)	237.2 (34–785, 269.7)	337.9 (17–3367, 548.8)
Horses (n = 122)	Sex	0 male/2 female	32 male/30 female/58 unknown
	Age in years, mean (range, Std. dev.)	Unknown	14.0 (0–30, 7.3)
	AST (normal < 500 U/l) in U/l (range, Std. dev.)	465.7 (360–571, 148.9)	646.3 (184–5234-1280, 672.9)
	ALT (normal < 43 U/l) in U/l (range, Std. dev.)	10 (10–10, 0)	16.1 (5–448, 49.4)

*For some pets the data age, sex, or transaminase level were not available (< 20%) and unfortunately could not be collected for data protection reasons.

**Figure 1:**
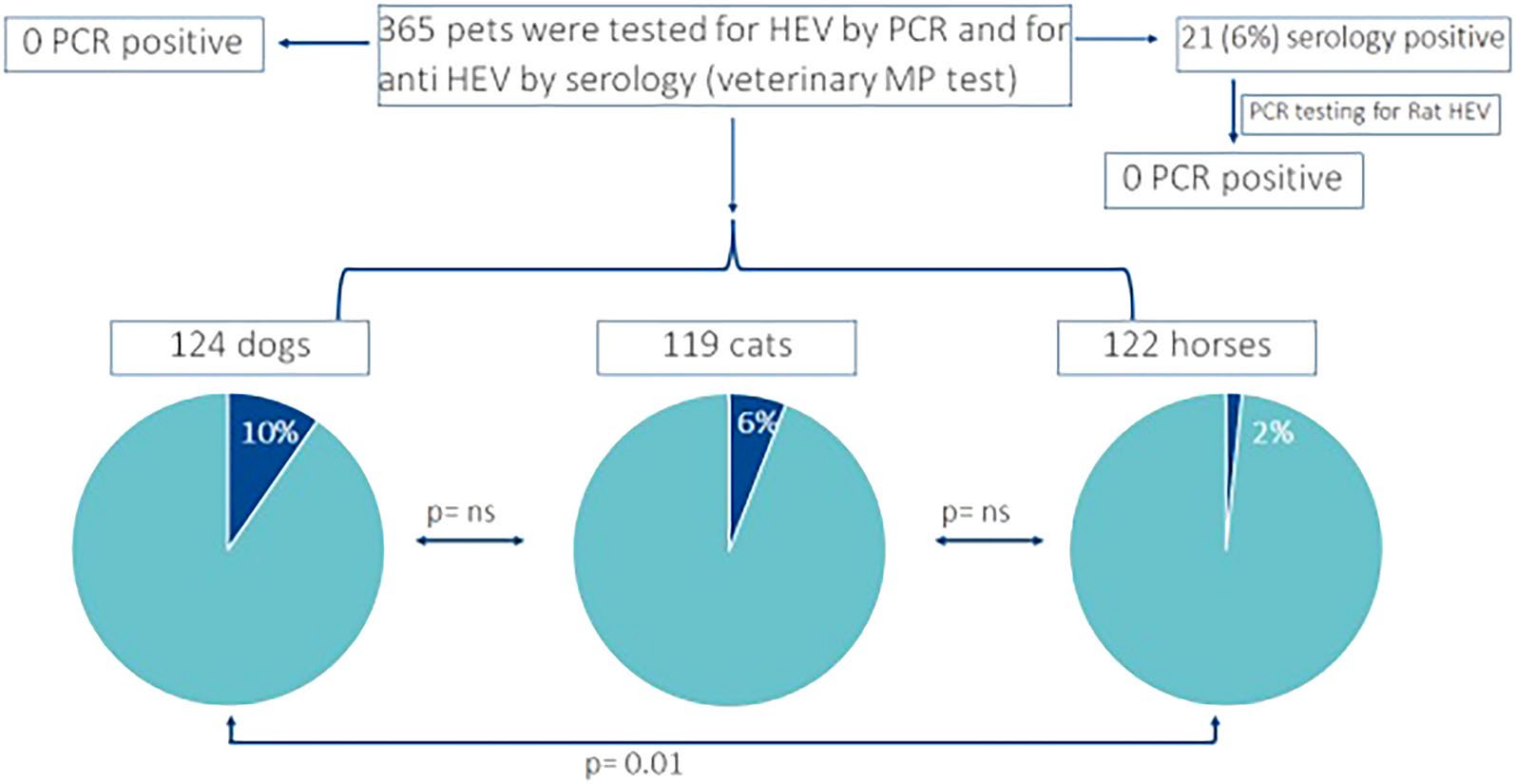
Results of serological and PCR testing.

The anti-HEV seroprevalence determined by the MP assay varied largely between 10% in dogs (12/124), 6% in cats (7/119), and 2% in horses (2/122) (Fig. 1). The difference between seroprevalence rates in dogs vs. horses (p = 0.01) reached statistical significance (Chi-square test).

**Figure 2:**
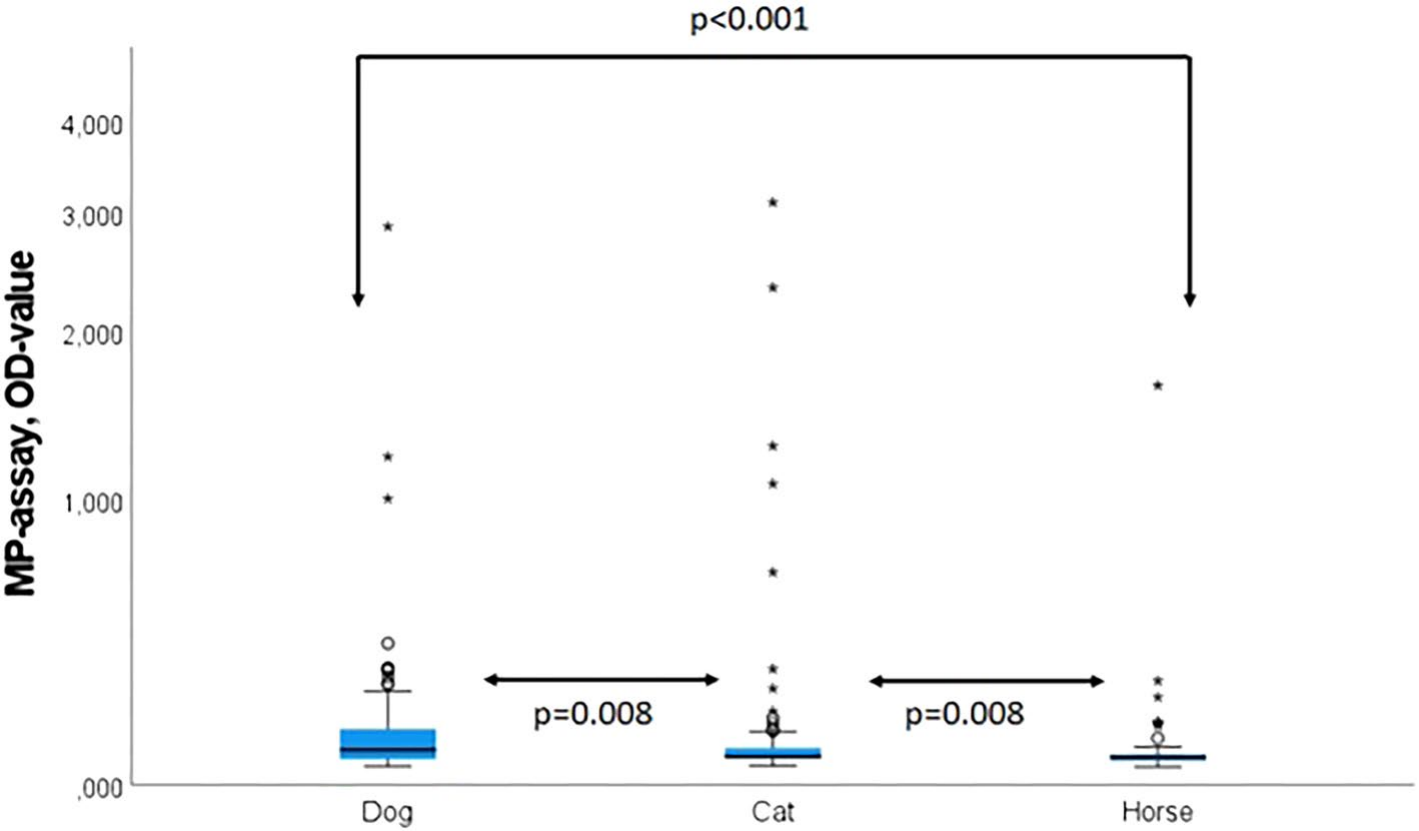
Levels of MP-assay OD values as a surrogate for anti-HEV levels.

Anti-HEV ELISA OD values were significantly higher in dogs in comparison to cats (p = 0.008) or horses (p < 0.001) and in cats compared to horses (p = 0.008, C, Mann–Whitney test) (Fig. 2). None of the animal serum samples tested PCR positive. All serologic-positive animals were re-tested with a broad range of RT-PCR covering tall genera of the *Orthohepevirinae* family but did not uncover any positive result.

An analysis of the samples by zip code of origin did not indicate a regional clustering of positive samples. The area of submission covers the whole of Germany and the positive samples came from Baden-Württemberg (n = 2), Bavaria (n = 4), Berlin (n = 2), Brandenburg (n = 2), Hessen (n = 2), Lower Saxony (n = 3), North Rhine-Westphalia (n = 3), Schleswig–Holstein (n = 2).

## Discussion

While the relevance of pork consumption for transmission of HEV genotype 3 infections in industrialized nations is well established, the role of domestic animals in the transmission of HEV genotype 3 infections in industrialized nations is still unclear.

The current study demonstrates that the risk of anti-HEV carriage and thus prior exposure to HEV in cats and dogs is between 6 and 10% Similar values of 9.9% for dogs and 2.2% for cats were found in a study from Spain^7^ using the same commercial ELISA. Such data, based on a veterinary serological assay, provide a clear picture of the relevance of HEV contacts in companion animals, whereas there is a wide variation in results for assays not specifically designed for animals: In dogs, the reported prevalences range from 19% up to 56.6%^8^ when the most often used Wantai ELISA was applied and 0% up to 23% for in-house ELISA (Table 1). A similar heterogeneous finding is seen in cats when using the Wantai ELISA (3.1%^9^ over 14.9%^10^ up to 32.3%^10^ or in-house ELISA from 2%^11^ over 11%^12^ up to 33%^13^ (Table 1).

The first finding of anti-HEV seropositive horses in Germany highlights the possible infection of equids with HEV. Seroprevalence from other horse studies shows a high variation from 0.4% up to 18.18% (Table 1). The lower seroprevalence in horses compared to dogs and cats may be explained by the fact that dogs and cats live directly in the same household as their owners and are carnivores, making transmission from the owner's meat products conceivable.

A so far underestimated risk could be the increasingly popular BARF diet (“Biologically Appropriate Raw Food”), i.e. feeding raw meat to dogs and cats, which could be a source of infection for parasites, bacteria, and viruses such as Feline and Canine Calicivirus and even then HEV^14^.

Although this pilot study provides a very good overview of the exposure of dogs, cats, and horses in Germany to HEV, no reliable conclusions can be made concerning individual dog, cat, or horse breeds because this information was missing in 38% of the animals. Prospective larger cohorts are needed if this aspect is to be studied in more detail.

While no significant difference regarding to transaminases could be found in HEV-negative vs. positive animals (Table 2), individual HEV-positive animals display increased liver enzyme values (Table 3). Although these could be interpreted as an expression of ongoing liver inflammation in the context of hepatitis E viremia that has just healed, there are numerous other possible causes. This includes Pancreatitis, Diabetes but also normal age-related changes, that reflect adaptations during the transition from young to adult individuals^15,16^.

**Table 3:** Characteristics of individual anti-HEV positive animals (MP-assay).

	Sex	Age	AST* (U/ml)	ALT** (U/ml)	Race
Dog 1	M	9	36	106	Belgian Shepherd
Dog 2	M	7	30	106	Miniature Schnauzer
Dog 3	F	Unknown	193	1525	Old English Bulldog
Dog 4	F	12	51	100	French Bulldog
Dog 5	M	10	45	108	Labrador Retriever
Dog 6	M	9	202	708	Unknown
Dog 7	M	14	47	104	Unknown
Dog 8	F	7	39	115	Mongrel
Dog 9	M	8	35	113	Labrador Retriever
Dog 10	M	9	90	492	Australian Shepherd
Dog 11	Unknown	14	221	974	Mongrel
Dog 12	Unknown	Unknown	Unknown	Unknown	Unknown
Cat 1	F	Unknown	30	45	Unknown
Cat 2	M	16	113	314	European Shorthaired Cat
Cat 3	F	Unknown	343	785	European Shorthaired Cat
Cat 4	F	Unknown	361	308	European Shorthaired Cat
Cat 5	F	14	25	34	European Shorthaired Cat
Cat 6	M	Unknown	21	56	Karthaeuser
Cat 7	Unknown	Unknown	42	118	Unknown
Horse 1	F	Unknown	360	10	Unknown
Horse 2	F	Unknown	571	202	Unknown

*AST normal values: < 65 U/l in dogs, < 47 U/l in cats, < 500 U/l in horses.

**ALT normal values: < 118 U/l in dogs, < 102 U/l in cats, < 43 U/l in horses.

An additional finding from the present study is—similar to all previous reports- that no viral RNA could be detected, which is a strong indication, that these animals can not be considered to be reservoir hosts so far. However, due to their close contact with humans, these animals can be regarded as sentinels, indicating that they share a common source of infection with their owners. Thus risk patients (e.g. transplant recipients) should be informed that dogs and cats—and to a minor degree horses—can indicate and determine risks of HEV exposure for humans.

## Data Availability

The datasets generated and/or analyzed during the current study are not publicly available, because the data were examined completely anonymously and public access would allow individual authors of this manuscript to identify the animals and their serological status. However, the data are available from the corresponding author on reasonable request. Due to anonymized testing, none of the authors of this paper can currently assign the serological results to individual animals with names and owners, and this anonymity should be preserved as far as possible.
