# Author Correction: Anti-HEV seroprevalence and rate of viremia in a German cohort of dogs, cats, and horses

**DOI:** 10.1038/s41598-023-47523-9

**Published:** 2023-11-24

**Authors:** S. Pischke, E. V. Knoop, M. Mader, L. Kling, A. Wolski, A. Wagner, K. Mueller, T. Horvatits, J. Stiller, K. Wisnewski, B. Kohn, J. Schulze zur Wiesch, M. H. Groschup, M. Eiden

**Affiliations:** 1 Department of Medicine, University Medical Center Hamburg-Eppendorf, Hamburg, Germany; 2 German Center for Infection Research (DZIF), Hamburg-Lübeck-Borstel and Heidelberg Partner Sites, Hamburg, Germany; 3 SYNLAB.Vet GmbH, Berlin, Germany; 4 Vetambulanz Hamburg, Hamburg, Germany; 5 Small Animal Clinic, School of Veterinary Medicine, Freie Universität Berlin, Berlin, Germany; 6 Small Animal Clinic, University of Leipzig, Leipzig, Germany; 7 Institute of Novel and Emerging Infectious Diseases, Friedrich-Loeffler-Institut, 17493 Greifswald-Insel Riems, Germany

Correction to: *Scientific Reports* 10.1038/s41598-023-46009-y, published online 07 November 2023

The Funding section in the original version of this Article was incomplete.

“Open Access funding enabled and organized by Projekt DEAL. This research was funded by the German Center for Infection Research (DZIF), Grant number DZIF TI 07.001, and the Else-Kröner-Fresenius-Stiftung, grant number 2019:EKFS10.”

now reads:

“Open Access funding enabled and organized by Projekt DEAL. This research was funded by the German Center for Infection Research (DZIF), Grant number DZIF TI 07.001, and the Else-Kröner-Fresenius-Stiftung, grant number 2019:EKFS10. We acknowledge financial support from the Open Access Publication Fund of UKE - Universitätsklinikum Hamburg-Eppendorf and DFG – German Research Foundation.”

The original Article has been corrected.

